# The Influence of *APOE* Genotype, DHA, and Flavanol Intervention on Brain DHA and Lipidomics Profile in Aged Transgenic Mice

**DOI:** 10.3390/nu15092032

**Published:** 2023-04-23

**Authors:** Anneloes Martinsen, Rasha N. M. Saleh, Raphael Chouinard-Watkins, Richard Bazinet, Glenn Harden, James Dick, Noemi Tejera, Matthew G. Pontifex, David Vauzour, Anne-Marie Minihane

**Affiliations:** 1 Norwich Medical School, University of East Anglia, Norwich NR4 7TJ, UK; 2 Clinical Pathology Department, Faculty of Medicine, Alexandria University, Alexandria 21526, Egypt; 3 Department of Nutritional Sciences, Faculty of Medicine, University of Toronto, Toronto, ON M5S 1A8, Canada; 4 Nutrition Analytical Service, Institute of Aquaculture, University of Stirling, Stirling FK9 4LA, UK

**Keywords:** Alzheimer’s disease, apolipoprotein E, docosahexaenoic acid, brain, flavonoids, phospholipids, PUFAs

## Abstract

The apolipoprotein E4 (*APOE4*) genotype is predictive of Alzheimer’s disease (AD). The brain is highly enriched with the omega-3 polyunsaturated fatty acid (n3-PUFA), docosahexaenoic acid (DHA). DHA’s metabolism is defective in *APOE4* carriers. Flavanol intake can play a role in modulating DHA levels. However, the impact of flavanol co-supplementation with fish oil on brain DHA uptake, status and partitioning, and according to *APOE* genotype is currently unknown. Here, using a humanised *APOE3* and *APOE4* targeted replacement transgenic mouse model, the interactive influence of cocoa flavanols (FLAV) and *APOE* genotype on the blood and subcortical brain PUFA status following the supplementation of a high fat (HF) enriched with DHA from fish oil (FO) was investigated. DHA levels increased in the blood (*p* < 0.001) and brain (*p* = 0.001) following supplementation. Compared to *APOE3,* a higher red blood cell (RBC) DHA (*p* < 0.001) was evident in *APOE4* mice following FO and FLAV supplementation. Although FO did not increase the percentage of brain DHA in *APOE4,* a 17.1% (*p* < 0.05) and 20.0% (*p* < 0.001) higher DHA level in the phosphatidylcholine (PC) fraction in the HF FO and HF FO FLAV groups, and a 14.5% (*p* < 0.05) higher DHA level in the phosphatidylethanolamine (PE) fraction in the HF FO FLAV group was evident in these animals relative to the HF controls. The addition of FLAV (+/− FO) did not significantly increase the percentage of brain DHA in the group as a whole. However, a higher brain: RBC DHA ratio was evident in *APOE3* only (*p* < 0.05) for HF FLAV versus HF. In conclusion, our data shows only modest effects of FLAV on the brain DHA status, which is limited to *APOE3.*

## 1. Introduction

The apolipoprotein E4 (*APOE4*) genotype is the strongest prevalent genetic determinant of Alzheimer’s disease (AD) risk with heterozygotes (*APOE3/E4*) and homozygotes (*APOE4/E4*) carriers at 3–4- and 12–15-fold increased risk, respectively, relative to the “wild-type” *APOE3/E3* genotype [1,2]. Despite this, the *APOE4* allele remains predictive rather than deterministic, with half of *APOE4/E4* never progressing to the development of AD [3]. This observed phenomenon suggests that environmental factors/circumstances may play a crucial role in *APOE4*-mediated AD development. Behavioural changes, such as dietary interventions, may modify or even prevent *APOE4*-mediated disease pathophysiology and ultimately cognitive decline.

The grey matter of the brain, particularly synaptic phospholipids, are docosahexaenoic acid (DHA)-rich, with several-fold higher DHA levels than in systemic tissue. The cognitive benefits associated with a higher DHA intake and status have been widely acknowledged in experimental animals [4] and in prospective cohort studies [5]. DHA is a crucial molecule for the neuronal structure and function [6,7]. It has anti-inflammatory and pro-resolving properties [8], improves nerve signalling [9], and reduces the amyloid burden [10]. More than 80% of brain DHA is esterified in the phospholipid pool [11]. DHA is highly concentrated in phosphatidylethanolamine (PE) followed by phosphatidylserine (PS), then phosphatidylcholine (PC), and finally in the phosphatidylinositol (PI) pool [12]. The distribution of DHA across these subclasses has been shown to be affected by the *APOE* genotype. Lower PE-DHA and PS-DHA levels were observed in older *APOE4* mice [13,14], which were corrected by DHA supplementation [14]. 

In fish oil (FO) intervention trials, the *APOE* genotype has been shown to affect DHA metabolism [15], including brain uptake and utilisation [7,16,17]. While responses were more favourable in *APOE3* carriers, *APOE4* carriers showed inconsistent and variable DHA levels in response to supplementation [18]. In some preclinical and clinical studies, the brain and blood DHA status was lower in *APOE4* carriers [18,19,20]. On the contrary, a higher uptake of DHA was seen in *APOE4* carriers in less advanced AD pathology compared to more advanced pathology, highlighting the importance of the timing of n-3 fatty acid supplementation in at-risk *APOE4* carriers. We have previously demonstrated an age-related decline in habitual cortex and hippocampal DHA levels, accompanied by diminished DHA-derived specialised anti-inflammatory and pro-resolving mediators (SPMs), which was more prominent in *APOE4* animals [21]. This was corrected by DHA supplementation with the complete restoration of brain DHA levels in *APOE4* animals [4]. 

There is growing evidence that the co-supplementation of FO with antioxidants can increase DHA status. The addition of selenium, tocopherols, vitamin A, vitamin B, vitamin C, and vitamin D all increased DHA blood levels [22,23,24,25]. Flavonoids, such as cocoa/tea-derived flavanols (FLAV) possess neuroprotective properties, with their anti-inflammatory properties often cited as mechanistically responsible [26,27,28]. Indeed, flavonoids have been shown to improve the hallmarks of AD pathology-mitigating abnormal glial cell activation, amyloid β deposition, and tau phosphorylation. Intriguingly, these effects may be more evident in non-*APOE4* carriers [29]. However, in the COSMOS-mind study, flavanol-rich cocoa extract alone did not benefit cognition [30]. Flavonoids may increase DHA uptake by preventing polyunsaturated fatty acid peroxidation, whilst the n-3 polyunsaturated fatty carbon chain facilitates the penetration of dietary flavonoids through cell membranes [31]. Therefore, the combined consumption of flavonoids and n-3 polyunsaturated fatty acids may enhance their therapeutic benefit. Despite this promising synergistic potential, no study has comprehensively investigated the impact of FLAV supplementation on habitual brain DHA and lipidomic profiles, their response to DHA intervention, and according to APOE genotype status. 

We hypothesize that the co-supplementation of fish oil with a flavanol-rich cocoa extract will modulate brain DHA in an *APOE* dependent fashion. A full lipidomic analysis was conducted to provide a more granular and holistic overview of DHA derivatives in different brain lipid pools. 

## 2. Materials and Methods

### 2.1. Study Approval

All the experiments were approved by the Animal Welfare and Ethical Review Body (AWERB); approval date: 17th February 2016 under the project licence code: PPL 70/8710). The experiments were conducted within the provisions of the Home Office Animals (Scientific Procedures) Act 1986. The reporting of the results complies with the guidance of the Animal Research: Reporting of In Vivo Experiments (ARRIVE) guidelines.

### 2.2. Animals Experimental Design and Dietary Treatments

120 male humanised *APOE3* (B6.129P2-Apoe^tm2(APOE*3)Mae^ N8) and *APOE4* (B6.129P2-Apoe^tm2(APOE*4)Mae^ N8) targeted replacement mice homozygous for the human *APOE3* or *APOE4* gene (Taconic, Germantown, NY, USA) were used in the experiments [32]. The mice were 10-months-old at the start of the experiments. They were maintained in a controlled environment as described previously [4]. In week 1, the animals were on a standard chow diet (RM3-P, Special Diets Services, Essex, UK). At week 2, the mice were assigned to five experimental groups and provided with one of the following diets (Research Diets Inc., New Brunswick, USA) for 22 weeks: a low fat diet (LF, 10 kcal%), a high fat diet (HF, 45 kcal%), a HF diet supplemented with fish oil (HF FO, 45 kcal% + 5.1 mg/g EPA/DHA; EPAX TGN, Oslo, Norway), a HF diet supplemented with cocoa flavanols (HF FLAV, 45 kcal% + 892 μg/g total cocoa flavanols from CHD-Q65ACTICOA-558; Barry Callebaut, Lebbeke-Wieze, Belgium) or a HF diet supplemented with a combination of both (HF FO FLAV). A full dietary composition is provided in the supplementary data (Supplementary Table S1). The doses of the n-3 polyunsaturated fatty acids and flavanols were chosen to be equivalent to a human diet. Using allometric scaling, the animals fed with FO received 9.2 mg/d EPA/DHA, equivalent to a 1.5 g/d human equivalent dose (HED), such as that found in a half-to-full portion of oily fish [33]. Similarly, the animals fed the FLAV diet received 3.1 mg/d of total cocoa flavanols of which 0.7 mg/d comprised catechin/epicatechin, which corresponds to a physiologically relevant HED of 508 mg/d of which 107 mg/d comprised catechin/epicatechin, which are the levels found in 33 g of FLAV-rich cocoa. All the diets were adjusted for caffeine (20 mg/d HED) and theobromine (157 mg/d HED), which are naturally present in cocoa. 

Food intake and body weight were recorded twice a week, three weeks prior to the intervention and for the duration of the intervention. Food pellets were replaced twice a week to avoid the oxidation of the bioactive compounds. At week 22, the mice were anaesthetised and blood was collected by cardiac puncture followed by the transcardiac perfusion of an ice-cold saline solution containing 10 IU/mL heparin (Sigma, Hertfordshire, UK). The plasma samples were isolated by centrifugation at 2000× *g* for 10 min and the samples were snap-frozen and stored at −80 °C. The brains were rapidly removed, bisected, snap-frozen, and stored at −80 °C. The half-brains were further dissected into corteces, hippocampi, subcortical regions, and cerebella, snap-frozen in liquid nitrogen and stored at −80 °C until further analysis. 

### 2.3. Lipid Extraction and Fatty Acid Analysis in Red Blood Cells and Feeds

The total lipids were extracted according to the method of Folch et al. [34] as described before [35]. Fatty acid methyl esters (FAME) were prepared by the acid-catalyzed transesterification of the total lipids [36,37]. The samples were dried overnight in a desiccator then methylation was carried out using 1.25M HCL in methanol. The FAME were separated using 50% saturated KCL then purified by elusion through SPE silica cartridges (Clean-up 203 Cusil 156, UCT) using isohexane: diethylether (95:5). The FAME were evaporated under oxygen-free nitrogen and re-suspended in 500 µL of iso-hexane. The purified FAME were then separated by gas-liquid chromatography using a ThermoFisher Trace GC 2000 (ThermoFisher, Hemel Hempstead, UK). On-column injection was carried out using a fused silica capillary column (ZBWax, 60 m × 0.32 × 0.25 mm i.d.; Phenomenex, Macclesfield, UK) and hydrogen as a carrier gas. The temperature gradient was from 50 to 150 °C at 40 °C/min, and then to 195 °C at 1.5 °C/min, and finally to 220 °C at 2 °C/min. Individual methyl esters were identified by comparison to known standards (Supelco 37-FAME mix; Sigma-Aldrich Ltd., Poole, UK) and by reference to published data [38]. The data were analysed using the Chromcard software package (Thermoquest Italia, Milan, Italy). Individual fatty acids were reported as percentages (% total fatty acids). This was calculated by measuring the relative area % of the chromatogram peak generated by the individual fatty acid and dividing it by the sum of all the relative area % of all the fatty acids in the chromatogram. The fatty acid content per g of tissue (nmol/g) was calculated using heptadecanoic acid (17:0) as the internal standard. Both measurements are reported as it is important to consider brain fatty acid content as a percentage of the total fatty acids and relative to the weight of the total lipids when interpreting the physiological meaning of the altered status [39].

### 2.4. Lipid Extraction and Fatty Acid Analysis in Plasma

Fatty acids in the plasma were extracted by a method modified from Folch et al. [34]. Briefly, the samples and the internal standard docosatrienoic ethyl ester (22:3n-3) were homogenised by vortex in a solution of 2:1 chloroform: methanol. The mixtures were kept at 4 °C overnight and brought to room temperature the next day. Potassium chloride (0.88% (*w*/*v*)) was added to the separate phases. The bottom organic phase containing the total lipid extract was transferred into new test tubes and dried down under a stream of nitrogen. Fatty acids were transmethylated at 100 °C for one hour with 14% (*v*/*v*) boron trifluoride methanol. The FAME were quantified on a Varian 430 gas chromatograph (Bruker, Billerica, MA, USA) equipped with a SP-2560 biscyanopropyl siloxane capillary column (100 m length × 0.25 mm diameter × 0.2 μm film thickness; Supelco, Belle-fonte, PA, USA). Details of the chromatography set-up method have been described previously [40].

### 2.5. Brain Lipidomics Profile

Lipids were extracted from the subcortical region samples (n = 10) via a modified Bligh–Dyer extraction [41], using methanol/water/dichloromethane in the presence of deuterated internal standards, using the Metabolon TrueMass^®^ Complex Lipid Panel (Rowarth, UK).

The extracts were dried under nitrogen and reconstituted in ammonium acetate dichloromethane: methanol. The extracts were transferred to vials for infusion-MS analysis, performed on a Shimadzu LC with nano PEEK tubing and the Sciex SelexIon-5500 QTRAP. The samples were analysed via both a positive and negative mode electrospray. The 5500 QTRAP was operated in MRM mode with a total of more than 1100 MRMs. Individual lipid species were quantified by taking the ratio of the signal intensity of each target compound to that of its assigned internal standard, then multiplying by the concentration of the internal standard added to the sample. The lipid class concentrations were calculated from the sum of all the molecular species within a class, and the fatty acid compositions were determined by calculating the proportion of each class comprised of individual fatty acids.

### 2.6. Statistical Analysis

Body weight and fatty acids data are presented as means ± SEM. The data analysis was performed using one-way and two-way ANOVAs. Standard diagnostic tests (e.g., the normality of residuals assessed using quantile–quantile plots and Shapiro–Wilk tests, outlier tests, high-leverage/influence data points tested using Cook’s distance) were used to verify that the data were appropriate for ANOVA analysis. Where necessary, transformations were applied to the response variance to ensure that the data complied with the ANOVA assumptions. If transformations could not be applied to ensure the normality of data, appropriate non-parametric statistical techniques (e.g., the Wilcoxon rank sum test) were used instead. Post hoc tests were carried out using the Tukey’s honest significant differences test. A multiple-test correction was performed. Along with a p-diet*genotype interaction, the analysis considered the effect of the genotype (independent of dietary group. p-genotype) and the effect of the diet (independent of genotype. p-diet) on the response to intervention. A statistical analysis was performed using R statistical software v.3.5.1 (R Foundation for Statistical Computing, Vienna, Austria). We utilised the brain: blood ratio of enrichment as a *pseudo* measure of brain DHA uptake [42].

## 3. Results

To determine whether the impact of a high fat diet on DHA concentrations in the brain can be modulated by a diet enriched with a combination of fish oil with flavanols and is influenced by *APOE* genotype, we analysed fatty acids’ profiles in the brain and blood pools of male *APOE3* and *APOE4* mice after a 22-week dietary intervention.

### 3.1. Effect of Diet on Brain, Plasma, and Red Blood Cell DHA Content

**Figure 1:**
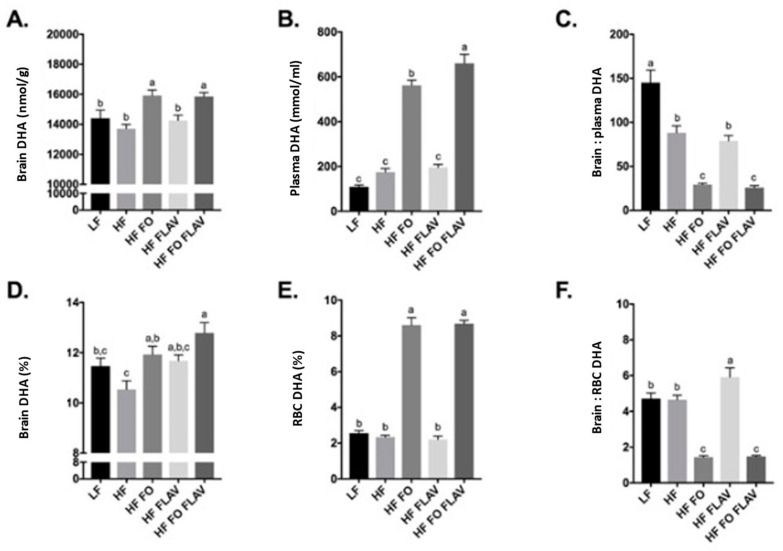
Effect of diet on brain, plasma, and red blood cells DHA content in mice, expressed in nmol/g of brain tissue (**A**), mmol/mL of plasma (**B**), and as % of total fatty acids (**D**,**E**). (**C**,**F**) are ratios of brain to plasma and brain to RBCs DHA concentrations respectively. Values are expressed as means ± SEM. One-way ANOVA Tukey’s post-hoc test adjusted *p*-values. a, b, c: columns (groups) with different letters are significantly different at *p* = 0.05, while columns sharing the same letters are not significantly different. Data of each dietary group was obtained from *APOE3* and *APOE4* mice combined. Number of mice per dietary group: LF n = 20, HF n = 21, HF FO n = 19, HF FLAV n = 19, and HF FO + FLAV n = 19.

First, the impact of FO, FLAV, and FO + FLAV on brain and blood DHA levels was analysed independently of *APOE* genotype. The brain and plasma DHA concentrations were higher for HF FO (*p* < 0.001) (Figure 1A,B). No additional effect of FLAV on brain DHA was observed (Figure 1A), while FLAV addition led to a higher plasma DHA concentration, *p* < 0.05 (Figure 1B). FO intervention reduced the brain: plasma DHA concentration ratio (Figure 1C), with the degree of enrichment in the plasma not reflected by a comparable increase in brain tissue (Figure 1A,B). Comparable trends were observed when the brain DHA (%) was compared to RBC (Figure 1D–F). A high-fat (HF) diet was not associated with changes in the DHA compared to a low-fat (LF) diet in the brain or the blood (Figure 1A,B,D,E); however, LF was associated with a higher brain: plasma DHA ratio.

### 3.2. Effect of APOE Genotype and Diet on Brain, Plasma, and RBC DHA Levels

**Figure 2:**
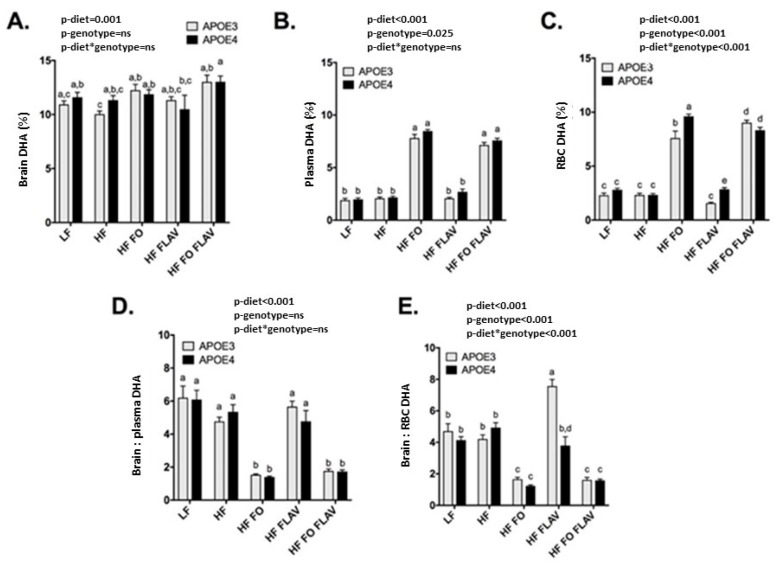
Effect of diet and genotype in the brain, plasma, and red blood cells DHA content of *APOE3* and *APOE4-TR* mice, expressed in % of total fatty acids (**A**–**C**) and ratios of brain: plasma (**D**) and brain: RBC DHA (**E**). Values are expressed as means ± SEM. a, b, c, d: columns (groups) with different letters are significantly different at *p* = 0.05, while columns sharing the same letters are not significantly different. Number of mice in each dietary and genotype group are: LF *APOE3* n = 11, LF *APOE4* n = 9, HF *APOE3* n = 13, HF *APOE4* n = 8, HF FO *APOE3* n = 11, HF FO *APOE4* n = 8, HF FLAV *APOE3* n = 10, HF FLAV *APOE4* n = 9, HF FO + FLAV *APOE3* n = 10, and HF FO + FLAV *APOE4* n = 8. P-diet*genotype: significance of diet and genotype interactive effect.

There was no effect of *APOE* genotype on the brain DHA status (Figure 2A). The *APOE4* mice had a higher DHA percentage in the plasma (*p* = 0.025 Figure 2B) and RBC (*p* < 0.001 Figure 2C) than the *APOE3* mice, independent of the dietary intervention. A diet–genotype interaction was evident for the RBC DHA (*p* < 0.001) with a higher percentage enrichment in the *APOE4* mice following the FO and FLAV intervention (Figure 2C), with comparable trends evident for the plasma DHA. We observed a genotype and diet-genotype interaction for the brain: RBC DHA ratio, with the higher ratio in *APOE3* versus *APOE4* animals (*p* < 0.001, Figure 2E), largely reflected by FLAV supplementation (*p* < 0.001, Figure 2E).

### 3.3. Impact of Diet and Genotype on DHA: AA Is More Evident in APOE4 Mice

Due to its impact on systemic and neuro-inflammatory status, the DHA: AA ratio was calculated in the blood and the brain. A higher RBC DHA: AA ratio was evident in *APOE4* compared to *APOE3* (*p* < 0.0001, Table 1). A diet–genotype interaction was observed in the DHA: AA ratio in the blood pools only (plasma and RBC, *p* = 0.021 and *p* < 0.000, respectively), with a significantly higher ratio in *APOE4* carriers in the HF FO, HF FLAV, and HF FO + FLAV diet groups compared to their corresponding *APOE3* dietary group.

A lipidomic analysis in the brain of *APOE3* and *APOE4* mice revealed no overall diet–genotype interaction on lipid classes or fatty acid concentrations. The total lipids DHA and arachidonic acids (AA) values did not differ between the genotypes (Supplemental Table S2).

### 3.4. Impact of Diet and APOE Genotype on Brain Phospholipid Subclasses’ DHA Content

The DHA concentration in the different phospholipid fractions was not different between the LF and the HF diet. In the *APOE3* mice, DHA supplementation (HF FO and HF FO + FLAV) increased the brain DHA in total phospholipids compared to the high-fat diet group (HF), being mostly reflected in the PC fraction (Table 2). Similar results were observed in the *APOE4* mice. Interestingly, a 17.1% (*p* < 0.05) and 20.0% (*p* < 0.001) higher DHA level in the PC fraction of the HF FO and HF FO + FLAV groups, and a 14.5% (*p* < 0.05) higher DHA level in the PE fraction of the HF FO + FLAV group was evident in the *APOE4* animals relative to the HF controls (Table 2).

The PC or PE fractions of arachidonic acid (AA) were not significantly different between the diet groups in the *APOE4* mice (Supplemental Table S3). In the *APOE3* mice, only the AA-PE fraction was reduced in the DHA-enriched groups in the *APOE3* animals (Supplemental Table S3).

## 4. Discussion

The present study provides the first comprehensive investigation of the impact of supplementation with FLAV with or without DHA on the brain lipidomic profiles and according to *APOE* genotype status, with a complex picture of DHA*FLAV*APOE interactions. Although the addition of FLAV did not significantly increase brain DHA in the group as a whole, FLAV led to an increase in the brain: RBC DHA ratio, which was limited to the *APOE3* animals. As expected, the brain DHA levels increased in the DHA-enriched dietary groups; however this was not influenced by the addition of FLAV. Following supplementation, higher blood (RBC and trend for plasma) DHA levels were observed in the *APOE4* mice relative to *APOE3.* This was accompanied by a lower brain: RBC ratio, which together highlights deficits in either DHA transport, storage, use, or turnover associated with the *APOE4* carrier status. However, in the *APOE4* animals a significant increase in DHA in both the brain PC and PE fractions was evident following DHA supplementation.

Although the size effect was modest, our research does highlight the potential of dietary flavanols as an approach to increase the brain DHA status independent of intake. Such strategies are of wide public health relevance, given concerns over fish and EPA/DHA sustainability [43], and given that the majority of the global population fail to meet dietary oily fish or EPA+DHA recommendations [44,45] and have a suboptimal EPA and DHA status [46]. It is particularly relevant to the estimated 25% of the world population who follow a vegetarian or vegan diet by choice or necessity, with negligible dietary EPA and DHA intake.

The number of people living with dementia worldwide is around 50 million [47]. With expanding and ageing populations, the incidence is predicted to almost triple by 2050. *APOE* genotype and obesity have been independently and interactively linked to AD risk, in humans and rodents [48]. In the present study we compared fatty acid and lipidomic profiles in the wild-type *APOE3* and with at-risk *APOE4* transgenic mice [2]. Animals were exposed to a high-fat diet to mimic human Western dietary practices, and in order to induce a human-like overweight phenotype with the associated insulin resistance and accelerated cognitive decline [48].

Observational studies have shown an association between DHA intake and reduced AD risk [49,50]. However, RCTs have shown inconsistent results, likely in part explained by the *APOE* genotype and AD stage. There is now relatively consistent evidence that the cognitive benefits associated with DHA supplementation are reduced in *APOE4* carriers [18,19,51]. Mechanistically, impaired BBB-mediated uptake and increased DHA oxidation and turnover are purported to be responsible. While there was an increase in the brain DHA level following fish oil supplementation in *APOE3* only, no difference between genotypes was evident for habitual DHA in the control (HF) group. We recently demonstrated a reduced habitual brain DHA level in 18-month-old female *APOE4* mice relative to younger females or age-matched *APOE3* males [21]. In addition, rodent studies performed on males and females have successfully demonstrated a genotype difference in response to DHA [42,52]. In the present study only male mice were used, which might explain some of the discrepancy. The cognitive deficits associated with *APOE4* are emerging as being more evident in females, potentially due to the additive effect of menopause and *APOE4*-carrier status on neurocognitive processes, with females also shown to benefit more from fish intake and DHA intake in old age [7,50]. Future research on DHA-*APOE* genotype- neurocognitive associations should consider sex as a potentially important mediating factor.

The capacity for DHA production in the brain is limited, with DHA predominantly derived from the systemic circulation, through dietary and systemic tissue pools. It has been shown that the plasma DHA metabolism is impaired in *APOE4* carriers [53]. We observed higher plasma and RBC DHA levels in the *APOE4* mice, in agreement with previous findings [42]. These genotype-mediated differences were reflected in the brain: RBC DHA values, with a higher ratio in *APOE3.* This may suggest a lower tissue uptake of DHA in *APOE4*, although a higher brain turnover cannot be precluded [17]. Furthermore, it indicates that supplementation with 1.5g DHA (HED) was not sufficient to raise DHA in the brain in the *APOE4* genotype. A higher dose of DHA (up to 3 g/d) may be needed in *APOE4* carriers, as has been suggested [52].

The combination of fish oil/DHA with other dietary compounds, such as flavonoids, is of particular interest, as it could help mitigate the negative impact of an *APOE4* genotype and potentially increase DHA bioavailability. In a 2010 analysis, green tea flavonoids (catechins) and fish oil had an additive effect on the inhibition of cerebral Aβ deposits in a mouse model of AD [54]. This combination of DHA and catechin also had an effect on cognitive performance (maze behaviour) in old mice, with the addition of catechin to the diet increasing brain DHA, possibly through the antioxidant properties of catechin [55]. In the present study the addition of cocoa FLAV did not significantly increase brain DHA levels. The lack of effect of catechin/epicatechin (FLAV) supplementation in our current study relative to the observations of Shirai and Suzuki [55] could be partly due to our use of a high-fat regime background diet (45% versus 6.5%). Interestingly, we observed that relative to LF feeding, the HF regime reduced the DHA concentration (in nmol/g) in the brain, as reflected in the lower brain: plasma DHA ratio, which could potentially buffer any physiological impact of FLAV on brain DHA levels. Furthermore, the brain region may have an impact on the size effect, with our analysis conducted in the subcortical region versus the whole brain in the previous analysis [55]. Somewhat consistent with a previous finding [55], we did observe a higher brain: RBC (%) ratio of DHA in the *APOE3*, but not the *APOE4* animals (Figure 2D), attributable to lower RBC DHA and a trend towards higher brain DHA (%). The aetiology of this finding is unknown, but is suggestive of an impact of FLAV on DHA biosynthesis, or retention/metabolism within the brain in the common *APOE3* genotype. This is worthy of further investigation, as it represents a potential means of increasing brain DHA without the need for increased intake.

The balance between DHA and AA is tightly regulated in the phospholipids of neuronal membranes and is crucial to ensure membrane fluidity, plasticity, and neurotransmission [52]. The DHA: AA ratio is also used in the diagnosis of MCI/AD pathology [56]. We recently demonstrated that old age and carrying an *APOE4* genotype decreased the brain DHA: AA ratio [21]. Interestingly, we did not observe any significant genotype effect in the brain DHA: AA ratio in the present study, which is likely attributable to the sex of the animals, with the effect more evident in females [4,7].

Less than 2% of fatty acids are present in the brain as free fatty acids. The majority are present as membrane phospholipids: PC, PE, PS, and PI. PS and PE are quantitatively the major brain DHA pools [39]. As ApoE is involved in brain phospholipid transport and metabolism, carrying an *APOE4* genotype may impair the structure of the neuronal membrane affecting DHA transport and downstream signalling pathways [57]. We observed that the higher total brain DHA in the DHA-enriched diet group in *APOE3* mice was reflected in the PC fraction. In *APOE4*, DHA supplementation increased DHA in both the PC and PE fraction, with the size effect for LPC not reaching significance. The addition of FLAV to the HF diet (with or without DHA) did not result in phospholipid-fraction enrichment. Although FO did not increase the percentage of brain DHA in *APOE4,* a 17.1% (*p* < 0.05) and 20.0% (*p* < 0.001) higher DHA level in the PC fraction of the HF FO and HF FO + FLAV groups, and a 14.5% (*p* < 0.05) higher DHA level in the PE fraction of the HF FO + FLAV group was evident in these animals relative to the HF controls. Flavonoids have been reported to increase the level of different phospholipids. For example, quercetin and naringenin increased PC and to a lesser extent PE in LPS-induced macrophages, which showed higher levels of longer chain and more polyunsaturated fatty acids (36–40 carbons) in PCs while no effect was observed in the PI and PS fractions. [58]. 

A limitation was that our analysis did not include the PS fraction, which is known to be a major anionic phospholipid class in neuronal membranes and particularly sensitive to DHA deprivation [59]. However, PE, which is a precursor of PS, was captured.

## 5. Conclusions

The current analysis shows an *APOE*-dependent change in brain and blood DHA levels, in response to fish oil and cocoa flavanols interventions. The *APOE4* genotype was associated with lower brain-DHA enrichment despite having higher blood DHA, compared to *APOE3*. An investigation into human bio-banked samples from human males and females is needed to confirm these findings and to help establish if *APOE4s* should be targeted with recommendations to optimise the DHA brain status. There was some evidence of a positive impact of flavanols on the brain-DHA status in *APOE3* only, which merits further investigation to confirm the findings and establish underpinning mechanisms.

## Figures and Tables

**Table 1:** Effect of diet and genotype on arachidonic acid (AA), DHA, and DHA: AA in plasma, red blood cells, and brain of *APOE3* and *APOE4-TR* mice after 22 weeks of intervention.

	*APOE3*					*APOE4*							
	LF	HF	HF FO	HF FLAV	HF FO FLAV	LF	HF	HF FO	HF FLAV	HF FO FLAV	Diet	Genotype	Interaction
Plasma (nmol/mL)													
20:4n-6 (AA)	857 ± 188	1595 ± 161	878 ± 95.7	2101 ± 137	1133 ± 144	850 ± 123	2224 ± 253	1008 ± 97.5	1887 ± 216	1268 ± 93.5	**<0.000**	NS	NS
22:6n-3 (DHA)	109 ± 12.8	149 ± 14.1	542 ± 43.3	183 ± 10	596 ± 62.5	108 ± 11.9	199 ± 27.2	582 ± 20	208 ± 26.1	724 ± 42.3	**<0.000**	**0.025**	NS
DHA:AA	0.14 ± 0.01	0.09 ± 00	0.64 ± 0.04	0.09 ± 00	0.54 ± 0.02	0.13 ± 0.01	0.09 ± 00	0.58 ± 0.06	0.11 ± 0.01	0.57 ± 0.06	**<0.000**	NS	**0.021**
Red Blood Cells (%)													
20:4n-6 (AA)	17 ± 0.74	19.1 ± 0.46	11.4 ± 0.57	15.2 ± 0.26	13.3 ± 0.37	18.3 ± 0.57	18.1 ± 0.65	13.7 ± 0.58	19.7 ± 0.46	11.8 ± 0.58	**<0.000**	**0.001**	**<0.000**
22:6n-3 (DHA)	2.48 ± 0.17	2.45 ± 0.12	7.74 ± 0.61	1.53 ± 0.07	8.42 ± 0.45	2.84 ± 0.14	2.34 ± 0.12	9.63 ± 0.2	2.84 ± 0.14	8.33 ± 0.26	**<0.000**	**<0.000**	**<0.000**
DHA:AA	0.14 ± 0	0.13 ± 0.01	0.67 ± 0.04	0.10 ± 00	0.63 ± 0.02	0.16 ± 0.01	0.13 ± 0.01	0.71 ± 0.04 ^a^	0.14 ± 0.01 ^b^	0.72 ± 0.05 ^c^	**<0.000**	**<0.000**	**<0.000**
Brain (%)													
20:4n-6 (AA)	8.48 ± 0.4	8.64 ± 0.28	7.55 ± 0.32	9.68 ± 0.37	8.45 ± 0.42	9.21 ± 0.32	9.53 ± 0.33	8.03 ± 0.33	8.95 ± 0.72	8.41 ± 0.39	**0.010**	NS	NS
22:6n-3 (DHA)	10.9 ± 0.33	10.0 ± 0.3	12.3 ± 0.54	11.3 ± 0.33	13.0 ± 0.63	11.6 ± 0.45	11.3 ± 0.43	11.9 ± 0.42	10.5 ± 1.3	13.0 ± 0.53	**0.001**	NS	NS
DHA: AA	1.3 ± 0.03	1.16 ± 0.03	1.63 ± 0.06	1.18 ± 0.04	1.55 ± 0.05	1.26 ± 0.03	1.19 ± 0.03	1.49 ± 0.04	1.11 ± 0.13	1.56 ± 0.04	**<0.000**	NS	NS

Values are expressed as means ± SEM. a, b, c significantly higher in *APOE4* compared to *APOE3*. Number of mice in each dietary and genotype group are: LF *APOE3* n = 11, LF *APOE4* n = 9, HF *APOE3* n = 13, HF *APOE4* n = 8, HF FO *APOE3* n = 11, HF FO *APOE4* n = 8, HF FLAV *APOE3* n = 10, HF FLAV *APOE4* n = 9, HF FO + FLAV *APOE3* n = 10, and HF FO + FLAV *APOE4* n = 8. Bold numbers show significant *p*-values.

**Table 2:** Diet effect on brain DHA phospholipid fractions in *APOE3* and *APOE4-TR* mice after 22 weeks of treatment.

Diet Group	LPC	LPE	PC	PE	PI	Total
*APOE3*						
LF	3.65 ± 0.66	4.55 ± 0.35	1049 ± 43.2	13,376 ± 802	9.66 ± 0.91	14,628 ± 802
HF	3.79 ± 0.23	4.35 ± 0.21	955 ± 41.2	12,355 ± 305	6.94 ± 0.97	13,519 ± 316
HF FO	3.53 ± 0.57	4.88 ± 0.34	1151 ± 29.8 **	14,732 ± 469	9.43 ± 1.04	16,142 ± 461 **
HF FLAV	2.50 ± 0.64	4.32 ± 0.30	932 ± 43.2	13,169 ± 451	7.71 ± 1.76	14,305 ± 473
HF FO FLAV	3.96 ± 0.42	5.29 ± 0.62	1089 ± 50.0	14,495 ± 455	8.57 ± 1.30	15,851 ± 448 *
*APOE4*						
LF	2.06 ± 0.53	4.59 ± 0.35	927 ± 32.4	13,090 ± 186	6.10 ± 1.29	14,199 ± 186
HF	2.43 ± 0.41	4.70 ± 0.37	941 ± 28.1	13,025 ± 295	7.10 ± 0.99	14,157 ± 310
HF FO	4.12 ± 0.71	5.09 ± 0.36	1102 ± 52.9 *	14,295 ± 508	7.82 ± 1.52	15,643 ± 468
HF FLAV	1.82 ± 0.50	4.32 ± 0.41	810 ± 106	11,801 ± 1536	5.11 ± 1.84	12,799 ± 1631
HF FO FLAV	3.47 ± 0.56	5.42 ± 0.77	1129 ± 34.2 **	14,896 ± 418 *	5.22 ± 0.97	16,269 ± 386

Values expressed in nmol/g of brain tissue. LF: low fat; HF: high fat; HF FO: high fat enriched in fish oil; HF FLAV: high fat supplemented in cocoa flavanols; HF FO FLAV: high fat combined with both fish oil and cocoa flavanols; LPC, lysophosphatidylcholine; LPE, lysophosphatidylethanolamine; PC, phosphatidylcholine; PE, phosphatidylethanolamine; PI, phosphatidylinositol. Total, total lipid in sample. Values are expressed as means ± SEM. * *p* < 0.05; ** *p* < 0.01 compared to HF. Number of mice in each dietary and genotype group are: LF *APOE3* n = 11, LF *APOE4* n = 9, HF *APOE3* n = 13, HF *APOE4* n = 8, HF FO *APOE3* n = 11, HF FO *APOE4* n = 8, HF FLAV *APOE3* n = 10, HF FLAV *APOE4* n = 9, HF FO + FLAV *APOE3* n = 10, and HF FO + FLAV *APOE4* n = 8.

## Data Availability

The data presented in this study are available on request from the corresponding author.

## Supplementary Materials

The following supporting information can be downloaded at: https://www.mdpi.com/article/10.3390/nu15092032/s1, Table S1: Fatty acid content in feeds; Table S2: Impact of diet and *APOE* genotype on brain neutral lipid AA and DHA content; Table S3: Diet effect on arachidonic acid phospholipid fractions in *APOE3* and *APOE4*-TR mice after 22 weeks of treatment.

## References

[1] Davidson Y., Gibbons L., Pritchard A., Hardicre J., Wren J., Stopford C., Julien C., Thompson J., Payton A., Pickering-Brown S.M. (2006). Apolipoprotein E ε4 Allele Frequency and Age at Onset of Alzheimer’s Disease. Dement. Geriatr. Cogn. Disord..

[2] Heffernan A.L., Chidgey C., Peng P., Masters C.L., Roberts B.R. (2016). The Neurobiology and Age-Related Prevalence of the epsilon4 Allele of Apolipoprotein E in Alzheimer’s Disease Cohorts. J. Mol. Neurosci..

[3] Michaelson D.M. (2014). APOE ε4: The most prevalent yet understudied risk factor for Alzheimer's disease. Alzheimer's Dement..

[4] Pontifex M.G., Martinsen A., Saleh R.N.M., Harden G., Fox C., Muller M., Vauzour D., Minihane A.-M. (2022). DHA-Enriched Fish Oil Ameliorates Deficits in Cognition Associated with Menopause and the APOE4 Genotype in Rodents. Nutrients.

[5] Jennings A., Cunnane S.C., Minihane A.M. (2020). Can nutrition support healthy cognitive ageing and reduce dementia risk?. BMJ.

[6] Arterburn L.M., Hall E.B., Oken H. (2006). Distribution, interconversion, and dose response of n−3 fatty acids in humans. Am. J. Clin. Nutr..

[7] Pontifex M., Vauzour D., Minihane A.-M. (2018). The effect of APOE genotype on Alzheimer's disease risk is influenced by sex and docosahexaenoic acid status. Neurobiol. Aging.

[8] López-Vicario C., Rius B., Alcaraz-Quiles J., García-Alonso V., Lopategi A., Titos E., Clària J. (2016). Pro-resolving mediators produced from EPA and DHA: Overview of the pathways involved and their mechanisms in metabolic syndrome and related liver diseases. Eur. J. Pharmacol..

[9] Tanaka K., Farooqui A.A., Siddiqi N.J., Alhomida A.S., Ong W.-Y. (2012). Effects of Docosahexaenoic Acid on Neurotransmission. Biomol. Ther..

[10] Lim G.P., Calon F., Morihara T., Yang F., Teter B., Ubeda O., Salem N., Frautschy S.A., Cole G.M. (2005). A Diet Enriched with the Omega-3 Fatty Acid Docosahexaenoic Acid Reduces Amyloid Burden in an Aged Alzheimer Mouse Model. J. Neurosci..

[11] Taha A.Y., Cheon Y., Ma K., Rapoport S.I., Rao J.S. (2013). Altered fatty acid concentrations in prefrontal cortex of schizophrenic patients. J. Psychiatr. Res..

[12] Norris C., Fong B., MacGibbon A., McJarrow P. (2009). Analysis of Phospholipids in Rat Brain Using Liquid Chromatography—Mass Spectrometry. Lipids.

[13] Sharman M.J., Shui G., Fernandis A.Z., Lim W.L.F., Berger T., Hone E., Taddei K., Martins I.J., Ghiso J., Buxbaum J.D. (2010). Profiling Brain and Plasma Lipids in Human APOE ε2, ε3, and ε4 Knock-in Mice Using Electrospray Ionization Mass Spectrometry. J. Alzheimer's Dis..

[14] Kariv-Inbal Z., Yacobson S., Berkecz R., Peter M., Janaky T., Lütjohann D., Broersen L.M., Hartmann T., Michaelson D.M. (2012). The Isoform-Specific Pathological Effects of ApoE4 in vivo are Prevented by a Fish Oil (DHA) Diet and are Modified by Cholesterol. J. Alzheimer's Dis..

[15] Chouinard-Watkins R., Rioux-Perreault C., Fortier M., Tremblay-Mercier J., Zhang Y., Lawrence P., Vohl M.C., Perron P., Lorrain D., Brenna J.T. (2013). Disturbance in uniformly^13^C-labelled DHA metabolism in elderly human subjects carrying the apoE ε4 allele. Br. J. Nutr..

[16] Yassine H.N., Rawat V., Mack W.J., Quinn J.F., Yurko-Mauro K., Bailey-Hall E., Aisen P.S., Chui H.C., Schneider L.S. (2016). The effect of APOE genotype on the delivery of DHA to cerebrospinal fluid in Alzheimer’s disease. Alzheimer's Res. Ther..

[17] Ebright B., Assante I., Poblete R.A., Wang S., Duro M.V., Bennett D.A., Arvanitakis Z., Louie S.G., Yassine H.N. (2022). Eicosanoid lipidome activation in post-mortem brain tissues of individuals with APOE4 and Alzheimer’s dementia. Alzheimer's Res. Ther..

[18] Yassine H.N., Braskie M.N., Mack W.J., Castor K.J., Fonteh A.N., Schneider L.S., Harrington M., Chui H.C. (2017). Association of Docosahexaenoic Acid Supplementation With Alzheimer Disease Stage in Apolipoprotein E ε4 Carriers. JAMA Neurol..

[19] Quinn J.F., Raman R., Thomas R.G., Yurko-Mauro K., Nelson E.B., Van Dyck C., Galvin J.E., Emond J., Jack C.R., Weiner M. (2010). Docosahexaenoic Acid Supplementation and Cognitive Decline in Alzheimer Disease: A randomized trial. JAMA.

[20] Chouinard-Watkins R., Conway V., Minihane A.M., Jackson K.G., Lovegrove J.A., Plourde M. (2015). Interaction between BMI and APOE genotype is associated with changes in the plasma long-chain–PUFA response to a fish-oil supplement in healthy participants. Am. J. Clin. Nutr..

[21] Martinsen A., Tejera Hernandez N., Vauzour D., Harden G., Dick J., Shinde S., Barden A., Mori T., Minihane A.M. (2019). Altered SPMs and age-associated decrease in brain DHA in APOE4 female mice. FASEB J..

[22] Trebble T., Arden N.K., Stroud M.A., Wootton S.A., Burdge G.C., Miles E.A., Ballinger A.B., Thompson R.L., Calder P.C. (2003). Inhibition of tumour necrosis factor-α and interleukin 6 production by mononuclear cells following dietary fish-oil supplementation in healthy men and response to antioxidant co-supplementation. Br. J. Nutr..

[23] De Cosmi V., Mazzocchi A., D’oria V., Re A., Spolidoro G.C.I., Milani G.P., Berti C., Scaglioni S., Giavoli C., Bergamaschi S. (2022). Effect of Vitamin D and Docosahexaenoic Acid Co-Supplementation on Vitamin D Status, Body Composition, and Metabolic Markers in Obese Children: A Randomized, Double Blind, Controlled Study. Nutrients.

[24] Song L., Zhou H., Yu W., Ding X., Yang L., Wu J., Song C. (2020). Effects of Phytosterol Ester on the Fatty Acid Profiles in Rats with Nonalcoholic Fatty Liver Disease. J. Med. Food.

[25] van Soest A.P.M., van de Rest O., Witkamp R.F., Cederholm T., de Groot L.C.P.G.M. (2022). DHA status influences effects of B-vitamin supplementation on cognitive ageing: A post-hoc analysis of the B-proof trial. Eur. J. Nutr..

[26] Vauzour D., Martinsen A., Layé S. (2015). Neuroinflammatory processes in cognitive disorders: Is there a role for flavonoids and n-3 polyunsaturated fatty acids in counteracting their detrimental effects?. Neurochem. Int..

[27] Jin H., Kim H.S., Yu S.T., Shin S.R., Lee S.H., Seo G.S. (2021). Synergistic anticancer effect of docosahexaenoic acid and isoliquiritigenin on human colorectal cancer cells through ROS-mediated regulation of the JNK and cytochrome c release. Mol. Biol. Rep..

[28] Pontifex M.G., Malik M.M.A.H., Connell E., Müller M., Vauzour D. (2021). Citrus Polyphenols in Brain Health and Disease: Current Perspectives. Front. Neurosci..

[29] Agarwal P., Holland T.M., James B.D., Cherian L.J., Aggarwal N.T., Leurgans S.E., Bennett D.A., Schneider J.A. (2022). Pelargonidin and Berry Intake Association with Alzheimer’s Disease Neuropathology: A Community-Based Study. J. Alzheimer's Dis..

[30] Baker L.D., Manson J.E., Rapp S.R., Sesso H.D., Gaussoin S.A., Shumaker S.A., Espeland M.A. (2022). Effects of cocoa extract and a multivitamin on cognitive function: A randomized clinical trial. Alzheimer's Dement..

[31] Sun C.Q., Johnson K.D., Wong H., Foo L.Y. (2017). Biotransformation of Flavonoid Conjugates with Fatty Acids and Evaluations of Their Functionalities. Front. Pharmacol..

[32] Zhu Y., Nwabuisi-Heath E., Dumanis S.B., Tai L.M., Yu C., Rebeck G.W., Ladu M.J. (2012). APOE genotype alters glial activation and loss of synaptic markers in mice. Glia.

[33] Minihane A.M. (2013). Fish oil omega-3 fatty acids and cardio-metabolic health, alone or with statins. Eur. J. Clin. Nutr..

[34] Folch J., Lees M., Sloane Stanley G.H. (1957). A simple method for the isolation and purification of total lipides from animal tissues. J. Biol. Chem..

[35] Pontifex M.G., Martinsen A., Saleh R.N.M., Harden G., Tejera N., Müller M., Fox C., Vauzour D., Minihane A. (2021). APOE4 genotype exacerbates the impact of menopause on cognition and synaptic plasticity in APOE-TR mice. FASEB J..

[36] Ghioni C., Bell J., Sargent J. (1996). Polyunsaturated fatty acids in neutral lipids and phospholipids of some freshwater insects. Comp. Biochem. Physiol. Part B Biochem. Mol. Biol..

[37] Christie W.W. (2003). Lipid Analysis.

[38] Ackman R.G., Connell J.J. (1980). Fish lipids. Advances in Fish Science and Technology.

[39] Lacombe R., Chouinard-Watkins R., Bazinet R.P. (2018). Brain docosahexaenoic acid uptake and metabolism. Mol. Asp. Med..

[40] Chouinard-Watkins R., Chen C.T., Metherel A.H., Lacombe R.S., Thies F., Masoodi M., Bazinet R.P. (2017). Phospholipid class-specific brain enrichment in response to lysophosphatidylcholine docosahexaenoic acid infusion. Biochim. Biophys. Acta (BBA)-Mol. Cell Biol. Lipids.

[41] Jensen S.K. (2008). Improved Bligh and Dyer extraction procedure. Lipid Technol..

[42] Vandal M., Alata W., Tremblay C., Rioux-Perreault C., Salem N., Calon F., Plourde M. (2014). Reduction in DHA transport to the brain of mice expressing human APOE4 compared to APOE2. J. Neurochem..

[43] Culliford A., Bradbury J. (2020). A cross-sectional survey of the readiness of consumers to adopt an environmentally sustainable diet. Nutr. J..

[44] Derbyshire E. (2019). Oily Fish and Omega-3s Across the Life Stages: A Focus on Intakes and Future Directions. Front. Nutr..

[45] Micha R., Khatibzadeh S., Shi P., Fahimi S., Lim S., Andrews K.G., Engell R.E., Powles J., Ezzati M., Mozaffarian D. (2014). Global, regional, and national consumption levels of dietary fats and oils in 1990 and 2010: A systematic analysis including 266 country-specific nutrition surveys. BMJ.

[46] Stark K.D., Van Elswyk M.E., Higgins M.R., Weatherford C.A., Salem N. (2016). Global survey of the omega-3 fatty acids, docosahexaenoic acid and eicosapentaenoic acid in the blood stream of healthy adults. Prog. Lipid Res..

[47] GBD 2016 Dementia Collaborators (2019). Global, regional, and national burden of Alzheimer’s disease and other dementias, 1990–2016: A systematic analysis for the Global Burden of Disease Study 2016. Lancet Neurol..

[48] Jones N.S., Rebeck G.W. (2018). The Synergistic Effects of APOE Genotype and Obesity on Alzheimer’s Disease Risk. Int. J. Mol. Sci..

[49] I Kosti R., I Kasdagli M., Kyrozis A., Orsini N., Lagiou P., Taiganidou F., Naska A. (2022). Fish intake, n-3 fatty acid body status, and risk of cognitive decline: A systematic review and a dose–response meta-analysis of observational and experimental studies. Nutr. Rev..

[50] Zhang Y., Zhuang P., He W., Chen J.N., Wang W.Q., Freedman N.D., Abnet C., Wang J.B., Jiao J.J. (2018). Association of fish and long-chain omega-3 fatty acids intakes with total and cause-specific mortality: Prospective analysis of 421 309 individuals. J. Intern. Med..

[51] Huang T.L., Zandi P.P., Tucker K.L., Fitzpatrick A.L., Kuller L.H., Fried L.P., Burke G.L., Carlson M.C. (2005). Benefits of fatty fish on dementia risk are stronger for those without APOEε4. Neurology.

[52] Chouinard-Watkins R., Vandal M., Léveillé P., Pinçon A., Calon F., Plourde M. (2017). Docosahexaenoic acid prevents cognitive deficits in human apolipoprotein E epsilon 4-targeted replacement mice. Neurobiol. Aging.

[53] Nock T.G., Chouinard-Watkins R., Plourde M. (2017). Carriers of an apolipoprotein E epsilon 4 allele are more vulnerable to a dietary deficiency in omega-3 fatty acids and cognitive decline. Biochim. Biophys. Acta (BBA)-Mol. Cell Biol. Lipids.

[54] Giunta B., Hou H., Zhu Y., Salemi J., Ruscin A., Shytle R.D., Tan J. (2010). Fish oil enhances anti-amyloidogenic properties of green tea EGCG in Tg2576 mice. Neurosci. Lett..

[55] Shirai N., Suzuki H. (2004). Effect of Dietary Docosahexaenoic Acid and Catechins on Maze Behavior in Mice. Ann. Nutr. Metab..

[56] Abdullah L., Evans J.E., Emmerich T., Crynen G., Shackleton B., Keegan A.P., Luis C., Tai L., LaDu M.J., Mullan M. (2017). APOE ε4 specific imbalance of arachidonic acid and docosahexaenoic acid in serum phospholipids identifies individuals with preclinical Mild Cognitive Impairment/Alzheimer’s Disease. Aging.

[57] Bazan N.G., Molina M.F., Gordon W.C. (2011). Docosahexaenoic Acid Signalolipidomics in Nutrition: Significance in Aging, Neuroinflammation, Macular Degeneration, Alzheimer's, and Other Neurodegenerative Diseases. Annu. Rev. Nutr..

[58] Conde T.A., Mendes L., Gaspar V.M., Mano J.F., Melo T., Domingues M.R., Duarte I.F. (2020). Differential Modulation of the Phospholipidome of Proinflammatory Human Macrophages by the Flavonoids Quercetin, Naringin and Naringenin. Molecules.

[59] Hamilton J., Greiner R., Salem N., Kim H.-Y. (2000). n−3 Fatty acid deficiency decreases phosphatidylserine accumulation selectively in neuronal tissues. Lipids.

