# Supplementary material for: The Influence of APOE Genotype, DHA, and Flavanol Intervention on Brain DHA and Lipidomics Profile in Aged Transgenic Mice
Source: Nutrients. 2023 Apr 23;15(9):2032. doi: 10.3390/nu15092032 (PMC10180974; doi:10.3390/nu15092032)
Supplement: Supplementary file 1 [file nutrients-15-02032-s001.zip › nutrients-2190039-supplementary.pdf]

Table S1: fatty acid content in feeds

Fatty acid	Chow diet (%)	Chow diet (mg/100g)	LF (%)	LF (mg/100g)	HF (%)	HF (mg/100g)	HF FO (%)	HF FO (mg/100g)	HF FLAV (%)	HF FLAV (mg/100g)	HF FO FLAV (%)	HF FO FLAV (mg/100g)
14:00	0.71	18.36	5.37	152.29	1.59	264.54	1.6	285.36	0.9	127.68	0.9	162.28
15:00	0.13	3.48	0.1	2.88	<LOQ	<LOQ	<LOQ	<LOQ	0.12	16.55	0.08	14.66
16:00	13.87	358.41	37.58	1065.37	39.22	6521.64	38.86	6938.19	38.4	5419.41	38.09	6873.82
18:00	2.75	71.02	10.58	299.91	5.1	848.34	5.13	915.08	6.81	961.12	6.83	1233.46
20:00	0.27	7.01	0.44	12.47	0.41	68.55	0.41	72.5	0.46	64.74	0.46	82.87
22:00	0.38	9.7	0.11	3.14	0.13	21.02	0.1	17.72	0.12	16.55	0.13	23.82
24:00	0.22	5.66	0.12	3.47	0.11	18.56	0.12	22.15	0.12	16.39	0.12	20.97
Total SFA	18.32	473.64	54.3	1539.54	46.56	7742.64	46.21	8251	46.93	6622.45	46.61	8411.88
16:1n-9	0.13	3.33	<LOQ	<LOQ	<LOQ	<LOQ	<LOQ	<LOQ	<LOQ	<LOQ	<LOQ	<LOQ
16:1n-7	0.81	20.89	0.22	6.25	0.19	31.24	0.15	26.78	0.21	29.34	0.22	39.7
18:1n-9	19.17	495.54	29.17	827.18	34.12	5672.72	33.56	5991.7	34.82	4913.94	33.9	6118.44
18:1n-7	1.56	40.43	0.79	22.29	0.77	127.82	0.77	137.54	0.79	110.96	0.82	148.84
20:1n-11	0.15	3.77	<LOQ	<LOQ	<LOQ	<LOQ	<LOQ	<LOQ	<LOQ	<LOQ	<LOQ	<LOQ
20:1n-9	1.4	36.22	0.19	5.43	0.21	35.22	0.24	43.7	0.21	29.34	0.26	47.24
20:1n-7	<LOQ	<LOQ	<LOQ	<LOQ	<LOQ	<LOQ	<LOQ	<LOQ	<LOQ	<LOQ	<LOQ	<LOQ
22:1n-11	1.38	35.63	0.19	5.43	0.21	35.22	<LOQ	<LOQ	<LOQ	<LOQ	<LOQ	<LOQ
22:1n-9	0.27	6.95	<LOQ	<LOQ	<LOQ	<LOQ	<LOQ	<LOQ	<LOQ	<LOQ	<LOQ	<LOQ
24:1n-9	0.25	6.39	<LOQ	<LOQ	<LOQ	<LOQ	<LOQ	<LOQ	<LOQ	<LOQ	<LOQ	<LOQ
Total MUFA	25.11	649.17	30.57	866.59	35.5	5902.23	34.72	6199.73	36.02	5083.58	35.21	6354.21
18:2n-6	47.59	1229.99	14.66	415.57	17.44	2899.7	16.99	3033.81	16.57	2338.06	16.11	2907.12
18:3n-6	<LOQ	<LOQ	<LOQ	<LOQ	<LOQ	<LOQ	<LOQ	<LOQ	<LOQ	<LOQ	<LOQ	<LOQ

20:2n-6	0.15	3.95	<LOQ	<LOQ	<LOQ	<LOQ	<LOQ	<LOQ	<LOQ	<LOQ	<LOQ	<LOQ
20:3n-6	<LOQ	<LOQ	<LOQ	<LOQ	<LOQ	<LOQ	<LOQ	<LOQ	<LOQ	<LOQ	<LOQ	<LOQ
20:4n-6	0.1	2.62	<LOQ	<LOQ	<LOQ	<LOQ	0.07	12.28	<LOQ	<LOQ	<LOQ	<LOQ
22:4n-6	<LOQ	<LOQ	0.16	4.52	0.18	29.92	0.07	12.49	0.17	24.42	0.17	30.34
22:5n-6	<LOQ	<LOQ	<LOQ	<LOQ	<LOQ	<LOQ	0.08	14.9	<LOQ	<LOQ	0.08	15.27
Total n-6 PUFA	47.84	1236.56	14.82	420.09	17.62	2929.62	17.21	3073.48	16.74	2362.48	16.36	2952.73
18:3n-3	5.64	145.79	0.32	9.00	0.32	53.4	0.32	56.39	0.31	43.6	0.32	57.82
18:4n-3	0.32	8.28	<LOQ	<LOQ	<LOQ	<LOQ	<LOQ	<LOQ	<LOQ	<LOQ	<LOQ	<LOQ
20:3n-3	<LOQ	<LOQ	<LOQ	<LOQ	<LOQ	<LOQ	<LOQ	<LOQ	<LOQ	<LOQ	<LOQ	<LOQ
20:4n-3	0.11	2.77	<LOQ	<LOQ	<LOQ	<LOQ	<LOQ	<LOQ	<LOQ	<LOQ	<LOQ	<LOQ
20:5n-3	0.98	25.23	<LOQ	<LOQ	<LOQ	<LOQ	0.37	65.85	<LOQ	<LOQ	0.36	65.56
21:5n-3	0.1	2.53	<LOQ	<LOQ	<LOQ	<LOQ	0.14	25.37	<LOQ	<LOQ	0.08	14.46
22:5n-3	0.16	4.1	<LOQ	<LOQ	<LOQ	<LOQ	0.07	12.89	<LOQ	<LOQ	0.08	14.46
22:6n-3	1.42	36.75	<LOQ	<LOQ	<LOQ	<LOQ	0.95	169.76	<LOQ	<LOQ	0.97	175.31
Total n-3 PUFA	8.72	225.45	0.32	11.92	0.32	53.4	1.85	330.27	0.31	43.6	1.82	327.61
Total PUFA	56.56	1462.01	15.13	429.09	17.94	2983.02	19.06	3403.75	17.05	2406.08	18.18	3280.34
**Oil (%)**	3.54		4.14		22.59		21.83		20.73		21.84	
**Moisture (%)**	11.55		6.13		6.35		6.57		5.86		6.18	

Table S2: Impact of diet and *APOE* genotype on brain neutral lipid AA and DHA content

Neutral and total lipids	Fatty acids	APOE3 (n=55)	APOE4 (n=42)	P-diet	P-genotype	P-diet:genotype
CE	18:1	39.08±3.87	28.49±2.61	NS	NS	NS
CE	20:4n-6 (AA)	9.77±0.9	7.41±0.79	**0.039**	NS	NS
CE	22:6n-3 (DHA)	25.34±2.96	14.5±1.48	**0.007**	**0.048**	NS
DAG	18:1	345.1±16.25	310.89±18.62	NS	NS	NS
DAG	20:4n-6 (AA)	85.11±3.43	79.42±4.19	NS	NS	NS
DAG	22:6n-3 (DHA)	28.25±1.5	25.64±1.78	0.090	NS	NS
FFA	18:1	393.35±11.42	389.74±9.37	NS	NS	NS
FFA	20:4n-6 (AA)	607.5±16.88	624.7±16.87	NS	NS	NS
FFA	22:6n-3 (DHA)	95.26±3.05	92.67±2.98	**0.000**	NS	NS
MAG	18:1	105.22±6.4	88.87±7.47	NS	NS	NS
MAG	20:4n-6 (AA)	24.07±1.26	21.48±1.69	NS	NS	NS
MAG	22:6n-3 (DHA)	0.81±0.19	0.72±0.21	NS	NS	NS
TAG	18:1	395.67±5.97	433.82±25.49	NS	NS	NS
TAG	20:4n-6 (AA)	39±0.49	41.59±0.81	**0.002**	**0.048**	NS
TAG	22:6n-3 (DHA)	61.41±1.05	61.96±1.14	**0.000**	NS	NS
Total	18:1	35370.82±1061.76	32656.96±1149.15	NS	NS	NS
Total	20:4n-6 (AA)	11090.14±213.99	10980.31±279.21	**0.050**	NS	NS
Total	22:6n-3 (DHA)	14832.43±261.23	14560.32±405.55	**0.001**	NS	NS

Supplemental table 2: Effect of genotype on brain neutral and total lipids in *APOE3* and *APOE4-TR* mice after 22-week of treatment, all dietary groups combined, expressed in nmol/g of tissue. AA, arachidonic acid; DHA, docosahexaenoic acid; CE, cholesteryl ester; DAG, diacylglycerol; FFA, free fatty acid; MAG, monoacylglycerol ; TAG, triacylglycerol; Total, total lipid in sample. Values are expressed as means ± SEM (n=42-55).

Table S3: Diet effect on arachidonic acid phospholipid fractions in *APOE3* and *APOE4*-TR mice after 22 weeks of treatment

**AA species**													
**Diet group**	**LPC[FA20:4]**	**LPE[FA20:4]**	**PC[FA20:4]**	**PE[FA20:4]**	**PI[FA20:4]**	**Total[FA20:4]**	**PC(16:0/20:4)**	**PC(18:0/20:4)**	**PC(18:1/20:4)**	**PE(16:0/20:4)**	**PE(18:0/20:4)**	**PI(16:0/20:4)**	**PI(18:0/20:4)**
**E3 LF**	7.11 ± 0.75 (11)	7.04 ± 0.47 (11)	1804 ± 176 (11)	8631 ± 617 (11)	139 ± 12.6 (11)	11329 ± 786 (11)	867 ± 92.4 (11)	627 ± 56.4 (11)	256 ± 26.3 (11)	340 ± 22.8 (11)	3188 ± 297 (11)	12.3 ± 1.05 (11)	112 ± 9.87 (11)
**E3 HF**	6.94 ± 0.42 (13)	7.58 ± 0.35 (13)	1791 ± 97.2 (13)	8960 ± 198 (13)	115 ± 6.79 (13)	11647 ± 261 (13)	878 ± 51.7 (13)	618 ± 31.4 (13)	240 ± 14.0 (13)	341 ± 7.43 (13)	3206 ± 115 (13)	10.3 ± 0.43 (13)	93.4 ± 5.85 (13)
**E3 HF FO**	6.07 ± 0.31 (11)	6.35 ± 0.32 (11)	1588 ± 124 (11)	7481 ± 219 (11)*	115 ± 8.83 (11)	9954 ± 273 (11)*	754 ± 66.5 (11)	570 ± 38.7 (11)	213 ± 18.9 (11)	297 ± 8.73 (11)	2853 ± 111 (11)	10.3 ± 0.71 (11)	93.3 ± 7.38 (11)
**E3 HF FLAV**	8.59 ± 0.65 (10)	7.17 ± 0.37 (10)	2078 ± 120 (10)	9147 ± 260 (10)	138 ± 14.0 (10)	12158 ± 236 (10)	1012 ± 63.3 (10)	722 ± 37.7 (10)	287 ± 19.7 (10)	356 ± 9.02 (10)	3535 ± 111 (10)	12.1 ± 1.10 (10)	113 ± 11.6 (10)
**E3 HF FO FLAV**	6.25 ± 0.82 (10)	7.06 ± 0.98 (10)	1885 ± 149 (10)	7482 ± 180 (10)*	120 ± 6.17 (10)	10286 ± 289 (10)	912 ± 76.1 (10)	662 ± 48.3 (10)	259 ± 22.8 (10)	305 ± 6.92 (10)	3055 ± 133 (10)	10.9 ± 0.58 (10)	97.5 ± 5.03 (10)
**E4 LF**	7.85 ± 0.55 (9)	7.35 ± 0.56 (9)	2019 ± 119 (9)	8409 ± 189 (9)	119 ± 12.0 (9)	11290 ± 199 (9)	983 ± 64.7 (9)	680 ± 36.5 (9)	304 ± 19.8 (9)	315 ± 9.51 (9)	3171 ± 81.2 (9)	10.2 ± 0.79 (9)	96.1 ± 10.1 (9)
**E4 HF**	7.10 ± 0.38 (8)	7.86 ± 0.55 (8)	2060 ± 176 (8)	8859 ± 187 (8)	122 ± 13.4 (8)	11905 ± 195 (8)	1012 ± 93.9 (8)	703 ± 52.1 (8)	289 ± 30.0 (8)	351 ± 7.70 (8)	3296 ± 91.3 (8)	10.8 ± 1.08 (8)	98.6 ± 11.1 (8)
**E4 HF FO**	6.83 ± 0.34 (8)**	6.62 ± 0.41 (8)	1703 ± 157 (8)	7992 ± 166 (8)	127 ± 10.6 (8)	10558 ± 324 (8)	811 ± 75.6 (8)	597 ± 55.2 (8)	242 ± 25.8 (8)	316 ± 6.48 (8)	2953 ± 110 (8)	11.9 ± 0.90 (8)	101 ± 8.45 (8)
**E4 HF FLAV**	6.50 ± 1.04 (9)	6.97 ± 0.69 (9)	1982 ± 177 (9)	7733 ± 1069 (9)	111 ± 17.2 (9)	10645 ± 1198 (9)	1007 ± 84.7 (9)	664 ± 53.7 (9)	261 ± 38.4 (9)	314 ± 41.9 (9)	2884 ± 409 (9)	9.74 ± 1.50 (9)	89.6 ± 14.0 (9)
**E4 FO FLAV**	6.45 ± 0.53 (8)	7.16 ± 0.86 (8)	1878 ± 152 (8)	7721 ± 258 (8)	123 ± 14.5 (8)	10506 ± 379 (8)	939 ± 81.3 (8)	622 ± 47.0 (8)	262 ± 23.3 (8)	302 ± 8.49 (8)*	2938 ± 137 (8)	11.5 ± 1.27 (8)	99.4 ± 11.9 (8)

Values expressed in nmol/g of tissue
